# Development of a Predictive Model for Large‐Volume Pleural Effusion After Separation Surgery in Patients With Spinal Metastatic Tumors

**DOI:** 10.1002/cnr2.70368

**Published:** 2025-11-19

**Authors:** Haochen Mou, Keyi Wang, Hao Qu, Yaling Jiang, Meng Liu, Xiaobo Yan, Xin Huang, Nong Lin, Zhaoming Ye

**Affiliations:** ^1^ The Second Affiliated Hospital of Zhejiang University School of Medicine Hangzhou Zhejiang People's Republic of China; ^2^ Orthopedics Research Institute of Zhejiang University Hangzhou Zhejiang People's Republic of China; ^3^ Clinical Research Center of Motor System Disease of Zhejiang Province Hangzhou People's Republic of China; ^4^ Zhejiang Key Laboratory of Motor System Disease Precision Research and Therapy Hangzhou Zhejiang People's Republic of China; ^5^ State Key Laboratory of Transvacuolar Implantation Devices Hangzhou China

**Keywords:** nomogram, pleural effusion, prognostic factor, spinal metastasis

## Abstract

**Background:**

Separation surgery followed by radiotherapy has emerged as a prevalent approach for managing spinal metastatic tumors. However, large‐volume postoperative pleural effusion (POPE) represents a challenging complication, as it potentially delays subsequent treatments and increases morbidity. This study aims to identify risk factors for large‐volume POPE and develop a predictive model for early identification to improve patient prognosis.

**Methods:**

This retrospective study analyzed 443 patients who underwent separation surgery for spinal metastases at our center between January 2014 and January 2022. High‐resolution CT‐based 3D modeling was utilized for accurate pleural effusion (PE) volume quantification. Variables including patient demographics, surgical details, and laboratory results were examined to identify risk factors associated with large‐volume POPE (≥ 1000 mL). A predictive nomogram was developed based on the multivariate logistic regression analysis.

**Results:**

Our findings indicated that advanced age, increased intraoperative blood loss, and decreased levels of preoperative serum albumin, postoperative serum protein, and hemoglobin were significant independent risk factors for large‐volume POPE. The predictive nomogram demonstrated high accuracy, with a mean AUC value of 0.953 for the training dataset and 0.927 for the testing dataset, indicating reliable predictability for identifying patients at high risk for large‐volume POPE.

**Conclusion:**

Our study identified independent risk factors for large‐volume PE following separation surgery in patients with spinal metastasis. The developed nomogram offers a practical tool for early identification of high‐risk groups, enabling timely and targeted interventions. By reducing the risk of large POPE, this approach may shorten hospitalization and accelerate the resumption of postoperative treatment, ultimately improving patient prognosis.

## 1 Introduction

Following lung and liver metastases, bone is the most common site of metastasis [1]. Up to 40%–70% of patients with malignant tumors can develop bone metastases, predominantly affecting the vertebral bodies as osteolytic lesions [2, 3]. While many metastases respond well to medical therapies such as hormone therapy, chemotherapy, and radiation, severe cases with epidural spinal cord compression (ESCC) necessitate surgical intervention because they can cause pain, spine instability, or even neurological dysfunction. Therefore, surgical treatment should be administered timely to relieve pain and restore movement and sensation, followed by adjuvant treatment such as radiotherapy or chemotherapy, depending on the patients' characteristics, tumor type, number of metastases, and other factors [4].

Separation surgery, first introduced by Laufer et al. in 2013, involves limited intralesional tumor resection and internal fixation, with subsequent thecal sac decompression [5]. This procedure is typically followed by radiotherapy to reduce residual tumor burden and enhance control. Due to its efficacy in spinal cord decompression and stable segmental instrumentation, separation surgery has become a leading approach for treating spinal metastatic tumors, facilitating subsequent adjuvant therapy [6].

Despite its advantages, pleural effusion (PE) is a commonly observed complication after this surgery [7, 8]. Recent studies reported an overall postoperative pleural effusion (POPE) rate of 50%–76% [9, 10, 11]. The clinical manifestations of PE can range from being asymptomatic to causing severe symptoms such as respiratory distress, shortness of breath, and low oxygen saturation [12]. In cases of large‐volume POPE, patients may develop hypoxemia, require prolonged intensive‐care stays, and even necessitate chest tube insertion for pleural drainage. While necessary, this procedure is often associated with various respiratory complications such as pneumothorax, infection, and re‐expansion edema. Importantly, multiple studies have shown that any complication delaying adjuvant radiotherapy beyond 4–6 weeks correlates with higher rates of local tumor progression and worse 1‐year survival in metastatic spine disease [13, 14, 15]. Chanbour et al. reported that each additional week of delay reduced local control probability by approximately 5% [16]. Early identification and mitigation of risk factors for large‐volume POPE therefore have direct oncologic significance.

The accurate assessment of POPE is paramount in the clinical management of patients [17]. Traditional methods of PE measurement, such as x‐ray and ultrasound, have limitations in precision and can only provide qualitative estimates of PE [18]. These limitations can lead to potential misjudgments in the severity of PE, which, in turn, may affect clinical decision‐making. Moreover, no predictive tool currently allows clinicians to estimate an individual patient's probability of developing large‐volume POPE and thus proactively tailor perioperative management. To address this need, we conducted a single‐center retrospective cohort study of 443 patients undergoing thoracolumbar separation surgery for metastatic spine disease. Our study introduces the use of 2‐mm thickness thin‐slice CT‐based 3D modeling of the chest CT data to conduct an accurate and quantitative method for PE measurement to explore the associated risk factors for large‐volume POPE and develop a predictive model. By doing so, clinicians can, at an early stage, initiate preemptive interventions in a potentially high‐risk group to reduce the risk of developing large‐volume POPE, allowing for a timely radiotherapy initiation to improve patient prognosis.

### 1.1 Ethical Statement

This retrospective study was approved by the ethics committee and institutional review board in the Second Affiliated Hospital, Zhejiang University School of Medicine (No. I20231116). The written informed consent was waived by our Ethics Committee due to the retrospective nature of the study.

## 2 Methods

### 2.1 Study Design and Patient Selection

**FIGURE 1:**
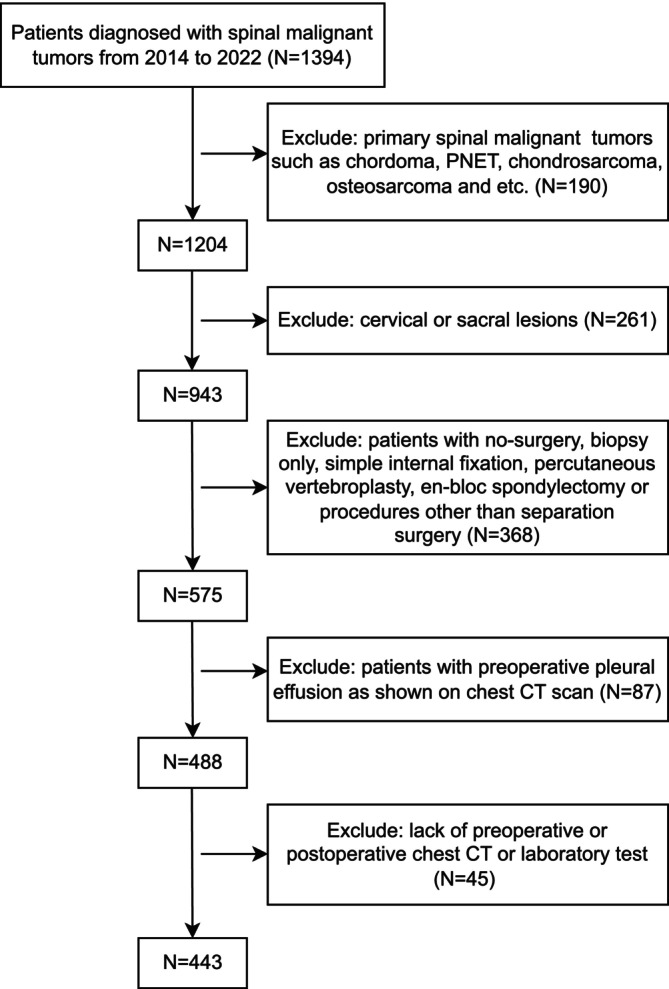
Flowchart shows the inclusion and exclusion criteria.

This study was conducted at the Second Affiliated Hospital, Zhejiang University School of Medicine, and was started on November 3, 2023. Patients who were admitted to our center and diagnosed with spine metastases between January 2014 and January 2022 were included for initial screening. Inclusion criteria encompassed individuals with spinal metastasis requiring separation surgery in the thoracolumbar spine with expected survival of more than 1 year. Indications for separation surgery are patients with obvious spinal cord compression graded as ESCC Grades II or III, or those exhibiting clear neurological symptoms corresponding to the involved segment. All included patients had an expected follow‐up duration of greater than 1 year. Exclusion criteria comprised patients with primary malignant spinal tumors, patients showing pleural invasion based on preoperative assessment, patients with preoperative PE, patients with surgically treated spinal metastases located in the cervical or sacral spine, and those for whom percutaneous vertebroplasty or simple internal fixation was employed instead of separation surgery. The detailed inclusion and exclusion process was shown in the flow diagram (Figure 1), and finally, 443 patients were included in this study. All patients undergoing separation surgery for spinal metastatic tumors at our institution are subjected to a standardized preoperative and postoperative monitoring protocol. As part of this protocol, chest CT scans are routinely performed preoperatively and postoperatively, with the latter typically conducted on the second or third day following surgery, after the removal of the drainage tube. This standardized practice, applied to all patients, regardless of cardiopulmonary symptoms, ensures comprehensive monitoring of preoperative and postoperative PE.

Expert consensus suggests that large‐volume thoracentesis refers to the removal of more than 1000 mL of pleural fluid [19]. Therefore, in this cohort, patients were divided into two groups: the large‐volume of POPE group, whose PE levels were equal to or greater than 1000 mL according to the 3D modeling calculation, and the moderate‐volume of POPE group, whose PE level was lower than 1000 mL. Data regarding patient characteristics (sex, age, BMI, lesion location [thoracic, lumbar, or multiple regions]), surgery (the use of artificial vertebrae, operation time, blood loss), and laboratory information (preoperative and postoperative serum hemoglobin level, serum protein level, serum C‐reactive protein level, serum albumin level, serum sodium level, serum potassium level, serum dehydrogenase, and glucose level) were collected from electronic medical records.

### 2.2 Explanatory Variable

After separation surgery, the accumulation of pleural fluid within the pleural space is predominantly exudate due to surgical stimulation and local inflammatory response. Hence, we included identifying factors that could contribute to exudative fluid formation for risk factor investigation. Laboratory tests were conducted for hemoglobin, total protein, albumin, potassium ion, lactate dehydrogenase, and glucose levels within 1 week before surgery and 1 day after surgery. Patient‐related factors, including gender, age, BMI, lesion location, reconstruction method, and intraoperative blood loss, were also included for association analysis.

### 2.3 3D Model Construction

**FIGURE 2:**
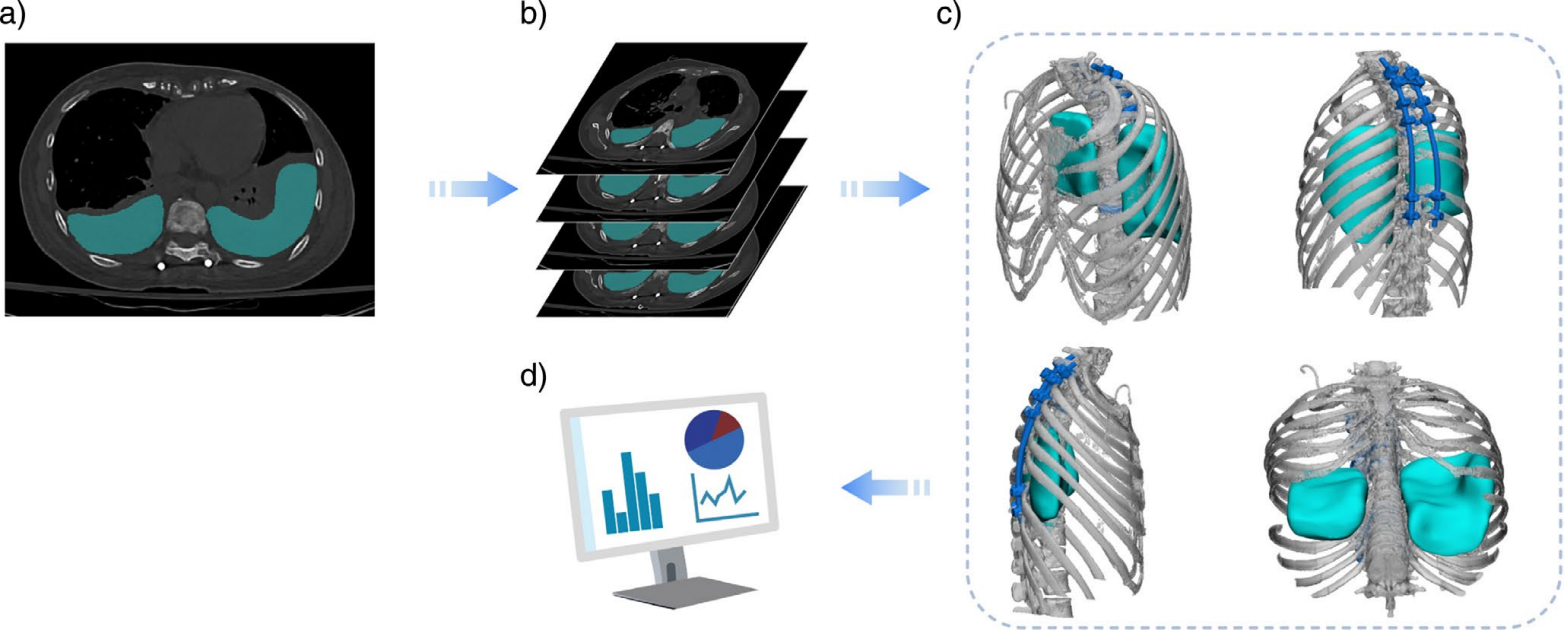
Three‐dimensional reconstruction and calculation of pleural effusion using thin‐sliced CT scans. (a) Import of chest CT scans into Mimics software to delineate pleural effusion across different sections based on Hounsfield Unit (HU) values. (b) Superimposition of multiple 2‐mm thickness CT layers to create a three‐dimensional representation of the chest cavity. (c) Visualization of the three‐dimensional model demonstrating the distribution of pleural effusion, viewable from multiple orientations. (d) Compilation and calculation of pleural effusion volume based on imported data.

To accurately quantify PE volume, this study employed high‐resolution CT images of patients' chests obtained post‐surgery for model reconstruction. Specifically, 2‐mm thin‐slice CT scans were utilized to ensure the highest precision in capturing the anatomical details necessary for accurate modeling. The imaging was performed using the Philips Brilliance 64‐slice CT scanner and the Philips Ingenuity Flex CT scanner. The DICOM format data of patients' chest CT data was first imported into the Mimics 20.0 software (Belgian company Materialize). The visualization of PE was achieved by leveraging the cross‐sectional CT images, and then a precise PE model was generated via the multilevel editing (multiple slice edit) command. Simultaneously, the software‐generated coronal and sagittal CT layers were utilized to meticulously delineate the edges of the PE model. Finally, the model was constructed, and the PE volume was calculated. Detailed procedures of PE calculation were illustrated in Figure 2.

### 2.4 Surgery

All patients in this study developed metastatic tumors in their thoracolumbar region and received separation surgery. The separation surgery is completed in the following steps: after exposing the spine through a posterior approach, pedicle screws were first inserted at the proximal and distal ends of the surgical segment. The vertebral lamina and facet joint articulations of the respective spinal segment were then excised. By approaching through the vertebral arch toward the posterior aspect of the vertebral body, a circumferential resection of tumor tissue around the dura mater, extending 5–8 mm, was performed. If the resection range of the vertebral body is less than 2/3 of the vertebral body, an appropriate amount of bone cement will be filled into the vertebral body based on the extent of vertebral resection. If the resection range of the vertebral body is equal to or larger than 2/3 of the vertebral body, we will opt for an artificial vertebral body to reinforce the anterior column. In cases where the tumor resection is minimal or demonstrates osteogenic changes, the use of bone cement may be omitted. Following thorough irrigation and hemostasis, the pedicle screw rod is connected, and two drainage tubes are placed, with subsequent layer‐by‐layer closure of the incision.

### 2.5 After Care

Patients will receive intravenous cefuroxime post‐surgery to prevent infection. Drainage tubes can be removed once the volume falls below 5 mL/h, after which antibiotics can be discontinued. High‐risk patients will receive subcutaneous low‐molecular‐weight heparin 48 h postoperatively. Close monitoring of symptoms like chest tightness, shortness of breath, and so forth is mandatory. Minimal‐volume POPE often requires no treatment; moderate‐volume POPE with symptoms may be managed pharmacologically; and large‐volume POPE with severe clinical symptoms may necessitate thoracentesis or drainage tube placement. All patients will receive postoperative radiotherapy for local control, with doses ranging from 30 to 60 Gy, depending on clinical condition and tumor characteristics, as determined by radiotherapists.

### 2.6 Statistical Analysis

Continuous variables were reported as means with standard deviations (mean ± SD) and were compared using the Student's *t*‐test or the Mann–Whitney *U* test, based on the adherence to a normal distribution. For categorical variables, the chi‐squared test or Fisher's exact test was applied as appropriate. Univariate logistic regression analysis was performed to evaluate potential factors associated with the incidence of large‐volume POPE. Variables that showed a marginal association with large PE (*p* < 0.1) were subsequently included in a multivariate logistic regression analysis. This analysis used a stepwise selection process to identify independent risk factors. A nomogram, derived from the *β* coefficients of each independent factor associated with large‐volume POPE, was constructed to quantify the probability of this condition. To evaluate the predictive accuracy of the nomogram, we utilized the *R package* “*rms*” and specifically employed the “*calibrate*” function for fivefold cross‐validation. In this approach, the dataset is randomly divided into five “folds” (subsets). Four folds are used for model training, and the remaining fold for testing. This approach was applied here to ensure robust model validation by averaging the predicted probabilities and actual probabilities across the five folds. The average predicted probabilities were then compared to the average actual probabilities to produce a final calibration curve. This method provides a reliable assessment of the nomogram's performance, confirming its applicability in predicting outcomes for the studied patient population. In addition, model accuracy was evaluated using receiver operating characteristic (ROC) analysis, with the area under the curve (AUC) serving as a measure of discriminatory ability—higher AUC values indicate better performance. All statistical tests were two‐sided, and a *p* value less than 0.05 is considered significant. Statistical analyses were performed using R Studio.

## 3 Results

### 3.1 Clinical Characteristics of Patients

A total of 443 patients were included in this study. The mean age of this cohort was 61 years; 259 were male and 184 were female. The metastatic tumors were primarily located in the thoracic region (*n* = 277), with 105 in the superior thoracic spine (T1–T4), 110 in the middle region (T5–T8), and 62 in the inferior region (T9–T12). There were 145 lesions located in the lumbar region and 21 cases involving mixed segments. Of all patients, the primary cancers were lung cancer (*n* = 139), multiple myeloma (*n* = 79), breast cancer (*n* = 35), liver cancer (*n* = 28), kidney cancer (*n* = 25), pancreatic cancer (*n* = 16), colorectal cancer (*n* = 16), and other types of cancers (*n* = 105). Clinical characteristics of patients were presented in Table 1.

**TABLE 1:** Clinical factors associated with the development of large‐volume POPE.

	[ALL]	PE < 1000 mL	PE ≥ 1000 mL	*p*
	*N* = 443	*N* = 397	*N* = 46	
Sex, *n* (%)				0.145
F	184 (41.5%)	170 (42.8%)	14 (30.4%)	
M	259 (58.5%)	227 (57.2%)	32 (69.6%)	
Median age, years (IQR)	61.0 [53.0, 68.0]	60.0 [52.0, 67.0]	66.0 [62.2, 72.0]	**< 0.001**
Median BMI (IQR)	22.6 [20.5, 24.6]	22.7 [20.6, 24.8]	22.3 [20.0, 23.7]	0.174
Lesion location, *n* (%)				0.123
Lumbar	145 (32.7%)	132 (33.2%)	13 (28.3%)	
Thoracic	277 (62.5%)	249 (62.7%)	28 (60.9%)	
Mixed lesion	21 (4.74%)	16 (4.03%)	5 (10.9%)	
Artificial vertebra, *n* (%)				0.872
No	346 (78.1%)	311 (78.3%)	35 (76.1%)	
Yes	97 (21.9%)	86 (21.7%)	11 (23.9%)	
Median op duration, h (IQR)	4.00 [3.30, 4.80]	4.00 [3.30, 4.80]	4.12 [3.60, 4.94]	0.54
Median blood loss, 100 mL (IQR)	8.00 [4.00, 12.0]	8.00 [4.00, 12.0]	12.5 [8.00, 16.0]	**< 0.001**
Median preop hemoglobin, g/L (IQR)	127 [114, 139]	127 [114, 139]	124 [106, 138]	0.22
Median preop protein, g/L (IQR)	67.6 [63.7, 72.2]	67.9 [64.0, 72.7]	64.9 [61.7, 68.8]	**0.001**
Median preop albumin, g/L (IQR)	38.0 [35.2, 40.4]	38.1 [35.3, 40.5]	37.0 [32.8, 39.8]	**0.021**
Median preop sodium chloride, g/L (IQR)	242 [238, 246]	242 [238, 246]	243 [238, 245]	0.453
Median preop potassium, mmol/L (IQR)	3.96 [3.72, 4.22]	3.97 [3.73, 4.24]	3.84 [3.63, 4.12]	**0.033**
Median preop dehydrogenase, U/L (IQR)	202 [166, 255]	202 [167, 253]	203 [163, 306]	0.65
Median preop glucose, mmol/L (IQR)	5.26 [4.69, 5.97]	5.20 [4.67, 5.94]	5.50 [4.87, 6.27]	0.085
Median postop hemoglobin, g/L (IQR)	102 [92.0, 111]	104 [93.0, 112]	92.5 [74.8, 99.8]	**< 0.001**
Median postop protein, g/L (IQR)	54.0 [50.1, 59.0]	54.6 [50.6, 59.6]	49.3 [45.5, 52.0]	**< 0.001**
Median postop albumin, g/L (IQR)	30.8 ± 3.94	30.9 ± 3.91	29.3 ± 3.95	**0.01**
Median postop sodium chloride, g/L (IQR)	241 [238, 245]	241 [238, 245]	243 [239, 246]	0.073
Median postop potassium mmol/L (IQR)	4.01 [3.78, 4.30]	4.02 [3.77, 4.30]	3.99 [3.84, 4.21]	0.976
Median postop dehydrogenase, U/L (IQR)	226 [186, 287]	225 [186, 285]	246 [177, 307]	0.775
Median postop glucose, g/L (IQR):	6.76 [5.61, 8.22]	6.76 [5.62, 8.21]	6.80 [5.59, 8.59]	0.714
Diagnosis, *n* (%)				0.294
Lung cancer	139 (31.4%)	130 (32.7%)	9 (19.6%)	
Myeloma	79 (17.8%)	69 (17.4%)	10 (21.7%)	
Breast cancer	35 (7.9%)	31 (7.8%)	4 (8.7%)	
Liver cancer	28 (6.3%)	22 (5.5%)	6 (13.0%)	
Kidney cancer	25 (5.6%)	23 (5.8%)	2 (4.3%)	
Pancreatic cancer	16 (3.6%)	13 (3.3%)	3 (6.5%)	
Colorectal cancer	16 (3.6%)	15 (3.8%)	1 (2.2%)	
Other	105 (23.7%)	94 (23.7%)	11 (23.9%)	

*Note:* Bold values indicate statistically significant differences (*p* < 0.05).

Abbreviations: F, female; IQR, interquartile range; M, male; op, operation; POPE, postoperative pleural effusion; postop, postoperative; preop, preoperative.

### 3.2 Risk Factors Associated With Potentially Large‐Volume POPE

The results of univariate and multivariate logistic regression analysis are presented in Tables 2 and 3. Univariate analysis revealed that older patients, lesion location, lesions involving more than 1 segment, intraoperative blood loss, preoperative and postoperative serum protein, postoperative serum hemoglobin, and postoperative serum albumin were associated with large‐volume POPE. The multivariate analysis identified that older patients (OR = 1.07, 95% CI: 1.02–1.13, *p* = 0.012), increased intraoperative blood loss (OR = 1.09, 95% CI: 1.02–1.16, *p* = 0.008), decrease in preoperative serum albumin level (OR = 0.83, 95% CI: 0.69–0.98, *p* = 0.032), decrease in postoperative serum hemoglobin level (OR = 0.94, 95% CI: 0.89–0.99, *p* = 0.023), and decrease in postoperative serum protein level (OR = 0.85, 95% CI: 0.75–0.95, *p* = 0.007) were independent risk factors for large‐volume POPE.

**TABLE 2:** Univariate analysis of risk factors for large POPE.

Explanatory variables	*β*	OR	95% CI	*p*
Sex				
Female		Reference		
Male	1.0986123	3	1.35, 7.61	< 0.001
BMI	−0.094311	0.91	0.82, 1.01	0.096
Age	0.0676586	1.07	1.03, 1.11	< 0.001
Lesion location				
Lumbar		Reference		
Thoracic	0.7371641	2.09	0.89, 5.75	0.116
Mixed lesion	1.9050882	6.72	1.76, 24.9	0.004
Artificial vertebra				
No		Reference		
Yes	0.0953102	1.1	0.45, 2.42	0.816
Operation duration (h)	0.10436	1.11	0.86, 1.41	0.398
Blood loss (100 mL)	0.0582689	1.06	1.01, 1.10	0.007
Preop hemoglobin (g/L)	−0.01005	0.99	0.98, 1.01	0.478
Preop protein (g/L)	−0.072571	0.93	0.87, 0.98	0.008
Preop albumin (g/L)	−0.072571	0.93	0.86, 1.00	0.055
Preop sodium chloride (g/L)	−0.01005	0.99	0.97, 1.02	0.633
Preop serum potassium (mmol/L)	−0.544727	0.58	0.25, 1.30	0.187
Preop glucose (mmol/L)	0.1655144	1.18	0.98, 1.39	0.062
Preop dehydrogenase (U/L)	0.0099503	1.01	0.99, 1.02	0.731
Postop hemoglobin (g/L)	−0.072571	0.93	0.91, 0.95	< 0.001
Postop protein (g/L)	−0.127833	0.88	0.84, 0.93	< 0.001
Postop albumin (g/L)	−0.105361	0.9	0.82, 0.99	0.025
Postop sodium chloride (g/L)	0.0487902	1.05	0.99, 1.11	0.155
Postop serum potassium (mmol/L)	0.0953102	1.1	0.48, 2.38	0.819
Postop glucose (g/L)	0.0676586	1.07	0.93, 1.21	0.337
Postop dehydrogenase (U/L)	0.0099503	1.01	1.00, 1.02	0.78

Abbreviations: PE, pleural effusion; POPE, postoperative pleural effusion; postop, postoperative; preop, preoperative.

**TABLE 3:** Multivariate logistic regression analysis of risk factors for large POPE.

Explanatory variables	*β*	OR	95% CI	*p*
Sex				
Female		Reference		
Male	0.91228271	1.52	0.64, 3.77	0.351
Age	0.06765865	1.07	1.02, 1.13	0.012
Blood loss (100 mL)	0.0861777	1.09	1.02, 1.16	0.008
Preop serum albumin (g/L)	−0.1863296	0.83	0.69, 0.98	0.032
Postop serum hemoglobin (g/L)	−0.0618754	0.94	0.89, 0.99	0.023
Postop serum protein (g/L)	−0.1625189	0.85	0.75, 0.95	0.007

Abbreviations: PE, pleural effusion; POPE, postoperative pleural effusion; postop, postoperative; preop, preoperative.

**FIGURE 3:**
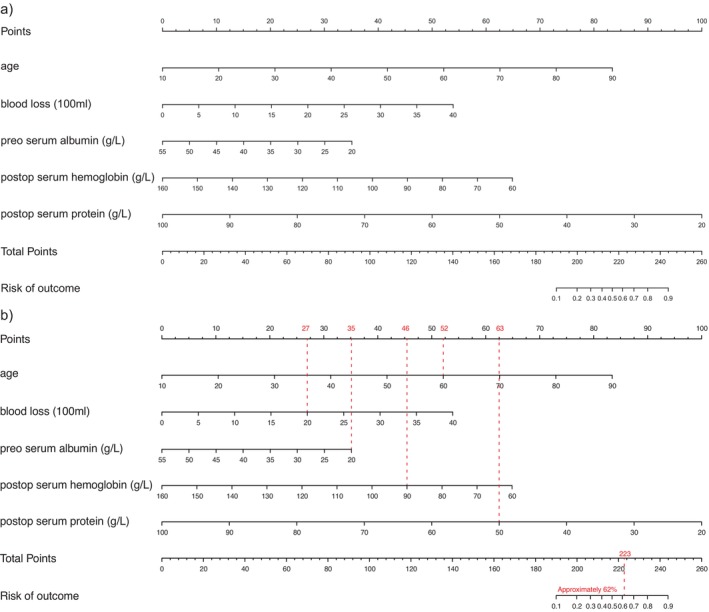
We created a nomogram that considers risk factors associated with large POPE. (a) The constructed nomogram displays predictive factors associated with the development of large‐volume POPE probability in patients with metastatic spine disease after separation surgery. (b) This nomogram demonstrates the probability of developing large POPE in a 60‐year‐old patient with intraoperative blood loss of 2000 mL, preoperative serum albumin level of 20 g/L, postoperative hemoglobin level of 90 g/L, and postoperative serum protein level of 50 g/L. The red dashed lines show that the patient received 52 points for age, 27 points for blood loss, 35 points for albumin level, 46 points for hemoglobin level, and 63 points for protein level; the total score, therefore, was 223 points, which corresponds to an approximately 62% chance that the patient will develop large‐volume of POPE.

The nomogram conducted in this study integrates the identified risk factors to provide a predictive tool for estimating the probability of developing large‐volume POPE (Figure 3a). To use the nomogram, each patient's risk factors are scored individually by drawing a vertical line from each risk factor to the “Points” row at the top of the nomogram. The “total points” are then calculated by summing up these points, which corresponds to the probability of developing large‐volume POPE, as indicated on the probability scale. For instance, consider a patient with the following characteristics: 60 years of age, intraoperative blood loss of 2000 mL, preoperative serum albumin level of 20 g/L, postoperative hemoglobin level of 90 g/L, and postoperative serum protein level of 50 g/L. The points calculated would be 223 points (52 + 27 + 35 + 46 + 63). According to the nomogram, this patient has an approximately 62% probability of developing large‐volume POPE (Figure 3b).

### 3.3 Predictive Accuracy

**FIGURE 4:**
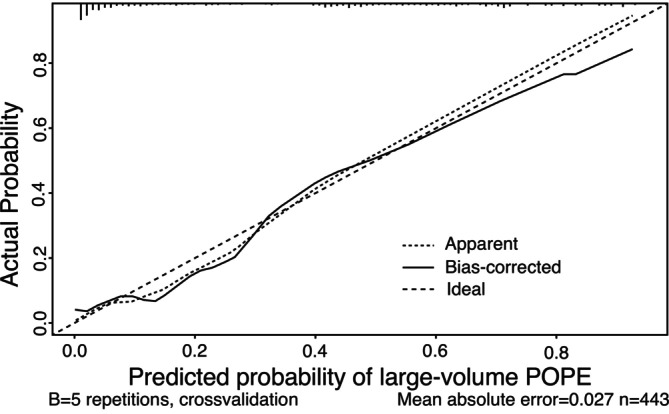
Calibration curve of the nomogram of large‐volume POPE. The *x*‐axis shows the predicted probability of large‐volume POPE, and the *y*‐axis shows the observed probability of large‐volume POPE.

Fivefold cross‐validation was used to assess the performance of the nomogram and ensure the predictive reliability of the model and mitigate the risk of overfitting. The model showed an average AUC of 0.953 (range: 0.939–0.970) for the training dataset and an average AUC of 0.927 (range: 0.861–0.979) for the testing dataset (Table 4). The calibration curve further demonstrates strong agreement between predicted and observed probabilities for large‐volume POPE. The bias‐corrected curve closely follows the ideal diagonal line across most probability ranges. A slight underestimation of risk was observed at the lower (< 0.3) and higher (> 0.6) ends of the prediction scale. However, this deviation is minimal and remains within clinically acceptable limits. Overall, the calibration results support the robustness and reliability of the predictive model (Figure 4).

**TABLE 4:** AUC values for both training and testing datasets derived from ROC analysis after fivefold cross‐validation.

AUC for training	Lower CI	Upper CI	AUC for testing	Lower CI	Upper CI
0.953	0.929	0.976	0.921	0.851	0.991
0.970	0.952	0.987	0.861	0.767	0.954
0.939	0.910	0.967	0.979	0.952	1.00
0.949	0.925	0.972	0.969	0.935	1.00
0.954	0.931	0.976	0.904	0.824	0.985

*Note:* The mean AUC for the training and testing datasets is 0.953 and 0.927, respectively.

## 4 Discussion

The management of PE in patients with metastatic spinal tumors after separation surgery is critical for ensuring that subsequent adjuvant therapies can be administered without delay. Epstein‐Peterson et al. in 2015 indicated that increasing time between surgery and radiotherapy initiation was associated with a greater risk of local recurrence [10]. Gong et al. in 2021 further suggested that a delay of over 1 month after surgery is associated with a higher incidence of tumor progression and subsequent decline in patient outcomes [13]. In addition, Blakaj et al. in 2021 also demonstrated the importance of minimizing delay from surgery to radiotherapy for spinal metastasis [15]. In addition to these oncologic considerations, PE itself has been shown to prolong hospitalization and recovery, which may indirectly contribute to such delays. For instance, Wiyono et al. demonstrated that patients with multiloculated PE experienced significantly longer hospital stays, particularly those with impaired lung function or more complex procedures. Taken together, these findings highlight that timely identification and management of PE is essential to prevent extended recovery periods that could postpone adjuvant therapy, thereby safeguarding local control and survival outcomes in patients with metastatic spinal tumors.

Our study quantitatively assesses the PE volume using high‐resolution chest CT 3D modeling, providing an objective baseline to categorize patients into risk groups. Rather than relying on therapeutic puncture as an indicator, we used quantitative volume, which we believe is more inclusive and representative of the entire cohort with POPE. The practice of chest tube insertion varies widely among clinicians and institutions, potentially confounding study results with practice patterns rather than patients' true condition. Therefore, a threshold of 1000 mL was set as the categorizing indicator. Based on our clinical practice, patients with 1000 mL of POPE are more likely to experience significant symptoms requiring intervention. Moreover, expert consensus defines large‐volume thoracentesis as the removal of over 1000 mL of pleural fluid [17]. Nevertheless, this does not negate the fact that individual responses can vary. This threshold serves more like a tool for risk stratification, and clinical decisions regarding PE management must always be made carefully.

In our study, we found that advanced age is associated with an increased risk of large‐volume POPE. Similar findings reported in several studies demonstrated that older age was an independent patient risk factor for large‐volume POPE [20, 21, 22]. In the elderly, the function of physiological reserve declines, which reduces their capacity to recover from the stresses of surgery. Concurrently, the elasticity of the lungs and chest wall decreases, which leads to impaired gas exchange and reduced clearance of fluid from the pleural space, increasing the risk of fluid accumulation. Furthermore, the prevalence of comorbid conditions, such as cardiovascular and renal disease, was more commonly seen in the elderly and can also contribute to fluid overload and the development of large POPE.

Our analysis identified increased intraoperative blood loss and decreased postoperative hemoglobin levels as independent predictors of large‐volume POPE. These findings are consistent with previous studies by Lu et al. and Xie et al., which also reported associations between hemoglobin concentration and POPE [22, 23]. The extent of blood loss during surgery directly impacts the patient's hemodynamic stability, potentially leading to fluid shifts and accumulation in the pleural space. Substantial blood loss can predispose patients to large POPE by disrupting the delicate balance of oncotic and hydrostatic pressures, thus facilitating the transudation of fluid into the pleural cavity. Similarly, postoperative hemoglobin levels serve as a marker for the overall severity of anemia after surgery. Anemia may impair oxygen delivery and trigger compensatory mechanisms such as increased cardiac output and volume retention, which can elevate pulmonary capillary pressure and indirectly promote PE. Patients with post‐surgical anemia, particularly due to significant intraoperative blood loss, can exacerbate hypoxia‐induced capillary leakage, further contributing to the risk of large POPE. We believe the association between lower hemoglobin levels and large POPE emphasizes the importance of meticulous intraoperative bleeding control and suggests a potential benefit for early management aimed at optimizing patients' preoperative and postoperative hemoglobin levels. Thus, the dual significance of intraoperative blood loss and postoperative hemoglobin offers compelling insight into the multifactorial nature of large POPE risk and highlights the importance of integrated perioperative management to mitigate this complication.

In our analysis, preoperative serum albumin and postoperative protein levels emerged as significant independent predictors for the development of large POPE. Such findings indicated the critical role of maintaining adequate protein homeostasis in the preoperative and postoperative periods. Serum proteins, especially albumin, are pivotal in sustaining oncotic pressure, which serves to balance fluid distribution across the vascular and extravascular spaces. A decline in postoperative protein levels can lead to an imbalance in this delicate system, facilitating the transudation of fluids into the pleural cavity, thereby increasing the risk of PE. This has been reported in multiple clinical contexts, including cardiac and hepatic surgery. A similar finding was reported by Peng et al., where they mentioned that malignant tumor patients with a decrease in serum protein had a higher risk of developing POPE and had a poorer prognosis [24]. Wulandary and Sofandi in 2024 also reported a case of a severe hypoalbuminemia patient with PE, where he noted that hypoalbuminemia can cause PE, particularly the transudative type, by reducing oncotic pressure, leading to fluid buildup [25]. We believe this finding highlights the need for meticulous monitoring of patients' serum protein and albumin levels pre‐ and post‐surgery as a routine part of patient care, and it calls for the integration of nutritional support strategies aimed at stabilizing or improving protein levels during hospitalization to better prevent large POPE.

Our nomogram extends these findings by providing a practical and predictive tool that integrates these risk factors, offering a risk assessment for patients with spinal metastasis undergoing separation surgery. Similar to what Wang et al. conducted for predicting postoperative pulmonary complications (PPCs) in hepatectomy patients, our model aims to enhance clinical decision‐making by identifying the potential high‐risk individuals for early and timely interventions [19]. Its high predictive accuracy, demonstrated through AUC values, affirms its potential utility in clinical practice. In addition to high discrimination, our model also demonstrated excellent calibration. The calibration curve showed strong agreement between predicted and observed probabilities across most risk levels. The bias‐corrected line closely followed the ideal diagonal reference line, suggesting minimal deviation between predicted and actual outcomes. There was a slight underestimation at the very low (< 30%) and very high (> 60%) predicted risk ends, where the model slightly underpredicted the actual rate. However, the differences were small and unlikely to affect clinical decision‐making.

In practical terms, clinicians can use the nomogram by entering readily available perioperative data (age, intraoperative blood loss, postoperative hemoglobin, preoperative albumin, and postoperative protein) to generate an individualized probability of developing large‐volume POPE. A patient with a predicted risk above 60%, for example, may warrant proactive strategies such as intensive chest imaging surveillance, early nutritional or transfusion support, and a lower threshold for pleural drainage to minimize treatment delays. Conversely, a patient with a predicted risk below 30% may be managed according to standard postoperative protocols without additional interventions. By stratifying patients in this way, the algorithm translates complex perioperative data into actionable decisions that can improve recovery and facilitate the timely initiation of adjuvant radiotherapy.

## 5 Limitation

Our study has several limitations. First, the retrospective nature of our study impedes the possibility of collecting comprehensive data on all variables that might influence the development of large POPE. For example, relevant factors such as preexisting comorbidities and prior systemic therapies were not collected for analysis in this dataset. This limitation may lead to the omission of relevant clinical and procedural variables that contribute to the risk of large POPE. Second, the study was conducted at a single tertiary institution, which may restrict the generalizability of findings to other settings with different clinical practices. In the future, external validation is necessary to assess the generalizability as well as evaluate the predictive accuracy of the model in multicenter or population‐based cohorts. In addition, despite utilizing 3D modeling for precise measurement of PE volume, the absence of a universally accepted definition for large‐volume PE introduces challenges in clinical interpretation. Individual patient factors can influence the risk associated with a given volume of PE. However, in clinical practice, a specific threshold has been correlated with increased potential for symptoms and the need for intervention. We believe setting a uniform threshold is crucial to balance the need for clear and objective management against the variability of individual patient responses.

## 6 Conclusion

This study identifies key independent risk factors for large‐volume PE following separation surgery in patients with spinal metastasis, including advanced age, increased intraoperative blood loss, and decreased levels of preoperative serum albumin, postoperative serum protein, and hemoglobin. The developed nomogram, with high AUC values in both training and testing datasets, offers a valuable tool for early identification of high‐risk patients, facilitating timely and targeted interventions.

## Author Contributions

**Haochen Mou:** funding acquisition (equal), investigation (equal), methodology (equal). **Keyi Wang:** data curation (equal), formal analysis (equal), methodology (equal), visualization (equal), writing – review and editing (equal). **Yaling Jiang:** investigation (equal), methodology (equal). **Meng Liu:** investigation (equal), methodology (equal), resources (equal). **Xiaobo Yan:** investigation (equal), methodology (equal), resources (equal). **Xin Huang:** investigation (equal), methodology (equal), resources (equal). **Nong Lin:** investigation (equal), methodology (equal), resources (equal). **Zhaoming Ye:** resources (equal), supervision (equal), validation (equal). **Hao Qu:** conceptualization (equal), project administration (equal), resources (equal), writing – original draft (equal), writing – review and editing (equal).

## Conflicts of Interest

The authors declare no conflicts of interest.

## Data Availability

De‐identified data can be available from the corresponding author upon reasonable request and with approval from the institutional ethics committee.
